# Genome-wide association study reveals novel loci for litter size and its variability in a Large White pig population

**DOI:** 10.1186/s12864-015-2273-y

**Published:** 2015-12-09

**Authors:** E. Sell-Kubiak, N. Duijvesteijn, M. S. Lopes, L. L. G. Janss, E. F. Knol, P. Bijma, H. A. Mulder

**Affiliations:** Animal Breeding and Genomics Center, Wageningen University, P.O. Box 338, 6700 Wageningen, AH The Netherlands; Topigs Norsvin Research Center B.V, P.O. Box 43, 6640 Beuningen, AA The Netherlands; Department of Molecular Biology and Genetics, Aarhus University, P.O. Box 50, 8830 Tjele, Denmark

**Keywords:** Double Hierarchical GLM, GWAS, Pigs, Residual variance, Total number born

## Abstract

**Background:**

In many traits, not only individual trait levels are under genetic control, but also the variation around that level. In other words, genotypes do not only differ in mean, but also in (residual) variation around the genotypic mean. New statistical methods facilitate gaining knowledge on the genetic architecture of complex traits such as phenotypic variability. Here we study litter size (total number born) and its variation in a Large White pig population using a Double Hierarchical Generalized Linear model, and perform a genome-wide association study using a Bayesian method.

**Results:**

In total, 10 significant single nucleotide polymorphisms (SNPs) were detected for total number born (TNB) and 9 SNPs for variability of TNB (varTNB). Those SNPs explained 0.83 % of genetic variance in TNB and 1.44 % in varTNB. The most significant SNP for TNB was detected on *Sus scrofa* chromosome (SSC) 11. A possible candidate gene for TNB is *ENOX1*, which is involved in cell growth and survival. On SSC7, two possible candidate genes for varTNB are located. The first gene is coding a swine heat shock protein 90 (*HSPCB = Hsp90*), which is a well-studied gene stabilizing morphological traits in *Drosophila* and *Arabidopsis*. The second gene is *VEGFA,* which is activated in angiogenesis and vasculogenesis in the fetus. Furthermore, the genetic correlation between additive genetic effects on TNB and on its variation was 0.49. This indicates that the current selection to increase TNB will also increase the varTNB.

**Conclusions:**

To the best of our knowledge, this is the first study reporting SNPs associated with variation of a trait in pigs. Detected genomic regions associated with varTNB can be used in genomic selection to decrease varTNB, which is highly desirable to avoid very small or very large litters in pigs. However, the percentage of variance explained by those regions was small. The SNPs detected in this study can be used as indication for regions in the *Sus scrofa* genome involved in maintaining low variability of litter size, but further studies are needed to identify the causative loci.

## Background

Conventional methods for studying the genetic architecture of complex traits focus on the level of those traits. In other words, the focus is on variation in trait level among genotypes. This implies that quantitative trait loci (QTL) can be defined as mean-controlling genes, as they affect the observed average phenotype of specific genotype. However, in many traits not only the mean is under genetic control, but also variation around the mean. Hence, genotypes not only differ in their average phenotypic level, but also in the variation around this average. In the following, we label this phenomenon as “phenotypic variability”, not to be confused with ordinary variability in average trait levels among genotypes. Therefore, development and application of statistical methods that allow studying phenotypic variability are required for a better understanding of the genetic architecture of complex traits [1–4].

The variation around the expected mean of a trait given its genotype, can be studied by analyzing the heterogeneity of residual variance across the observations [1]. It has been found that genotypes differ in residual variance [5]. Empirical evidence was found that the residual variance has a genetic component for litter size in rabbits [6], birth weight in pigs [7, 8], body weight in Atlantic salmon [9] and milk production traits in dairy cattle [10–12]. In addition, some studies have reported QTL that are associated with phenotypic variability, so-called vQTL [3]. The presence of vQTL in a population can indicate the existence of unmodeled interaction associated with the locus [3, 13, 14]. Three types of interactions can be distinguished, in which vQTL could be involved: interaction between the genes (epistasis) [3], interaction between the gene and known/unknown environmental factors [15, 16] or parallel presence of both of those interactions [3]. The fourth type of vQTL is one that controls the variance of a trait [17]. Several studies have reported vQTL in plant (maize [18], *Arabidopsis thaliana* [13]) and animal species (*Drosophila melanogaster* [5], rat [19], dairy cows [20] and in humans [15, 21]). One of the most well-studied genes involved in buffering effects of genetic and environmental factors is heat-shock protein 90 (*Hsp90*). This gene was described in *Drosophila* and *Arabidopsis* as a gene stabilizing developmental and morphological traits [22–24].

In this study, we focus on litter size and its variability in a Large White pig population. Many studies have reported single nucleotide polymorphisms (SNP) and QTL for the mean litter size of a genotype, and such QTL have been found on all *Sus scrofa* chromosomes (SSC) except SSC11, SSCX and SSCY (PigQTLdb, [25]). However, on top of the genetic variance in litter size, there is considerable residual variation between sows for litter size (total number born). Most issues are caused by extremely large litters (e.g. litter sizes greater than 25 piglets), which exceeds the physiological capacity of the sow to provide for the litter during gestation and post-farrowing. Sows with the large litters can experience welfare issues such as high energy demands during gestation [26] and shoulder sores during lactation [27, 28]. Moreover, these extreme litter sizes reduce also welfare and survival of the piglets pre-farrowing and until weaning. In current pig breeding, the goal is towards more sustainable production that will increase piglet survival regardless of increasing litter size [29–33]. Decreasing the variation in litter size between sows could lead to more sustainable breeding in terms of lower mortality of piglets and easier to manage sows. Therefore, it is desirable to reduce the variation in litter size from both an economic and an animal welfare perspective. Moreover, the detection of genes that buffer environmental factors, and thus decrease the variability of TNB is highly desirable. Thus far, Sorensen and Waagepetersen [34], Rönnegård et al. [2] and Felleki et al. [35] showed on the same dataset that variability in total number born in pigs is heritable. However, no study reported genomic regions associated with litter size variability or other traits in pigs [36]. A genome-wide association study (GWAS) for variability of litter size would give more insight in the genetic and biological control of variability in litter size.

The main objective of this study was to identify SNPs associated with litter size (TNB) and its variation (varTNB), through a multi-SNP GWAS applying a Bayesian method. In total, 10 SNPs were detected for TNB and 9 SNPs for varTNB. The most significant SNP for TNB was detected on SSC11 and for varTNB on SSC7. A possible candidate gene for TNB on SSC11 is *ENOX1*, which is involved in cell growth and survival. On SSC7, two possible candidate genes for varTNB are located. The first is a gene coding a swine heat shock protein 90 (*HSPCB = Hsp90*), which is a well-studied gene stabilizing morphological traits in *Drosophila* and *Arabidopsis*. The second is *VEGFA*, which is activated in angiogenesis and vasculogenesis in the fetus. We also found a positive genetic correlation between TNB and its variance, indicating that single-trait selection for TNB will increase the varTNB. To our knowledge, this is the first study reporting SNPs for TNB on SSC11 and SNPs associated with varTNB in pigs.

## Results and discussion

The main objective of this study was to detect regions associated with litter size (total number born, TNB) and its variation (varTNB) in a Large White pig population using a genome-wide association study (GWAS). Prior to the GWAS, the phenotypes for the association study had to be obtained. Therefore, as the first step, a Double Hierarchical GLM (DHGLM) was used to estimate variance components and estimated breeding values (EBV) of TNB and varTNB. Second, the EBV obtained with DHGLM were deregressed. Finally, the deregressed EBV were used as phenotypes in the GWAS. In this section, we present and discuss all the results that lead to detecting regions associated with TNB and varTNB.

### DHGLM analysis of litter size and its variation

Table 1 shows estimates of variance components and heritability obtained from the univariate analysis of TNB, which are within the range known from the literature, where heritability estimates for TNB range from 0.10 to 0.16 [32, 33, 37–39]. The variance components estimated with univariate analysis of TNB were used as starting values for DHGLM.

**Table 1:** Genetic parameters (with SE) from a conventional univariate analysis of litter size (TNB)

Estimate	TNB
Additive genetic variance	1.31 (0.04)
Permanent sow variance	0.87 (0.03)
Residual variance	7.14 (0.02)
Heritability	0.14 (0.004)

The variance components and heritability for TNB from the DHGLM presented in Table 2 are also in the expected range [32, 33, 37–39]. For varTNB the estimate of additive genetic variance and heritability (Table 3) are lower than previously reported for this trait [2, 35]. It is worth noticing, that this heritability is a measure of the reliability of EBV for varTNB based on single observations; it does not reflect the magnitude of the genetic variance in varTNB [40].

**Table 2:** Variance components (with SE) estimated in Large White sows for litter size (TNB) and residual variance of litter size (varTNB) using a Double Hierarchical GLM (DHGLM) and for mean litter size per sow (meanTNB) and log-transformed variance of the TNB per sow (log (var (TNB)) using conventional bivariate analysis

	DHGLM		Conventional	
Estimates	TNB	varTNB	meanTNB	log(var(TNB))
Additive genetic variance	1.18 (0.04)	0.03 (0.003)	1.23 (0.04)	0.04 (0.004)
Permanent sow variance	0.69 (0.02)	0.15 (0.004)	−^a^	−^a^
Residual variance	6.5 (0.02)^b^	1.88 (0.01)^b^	11.8 (0.15)	3.78 (0.03)
Heritability	0.14 (0.003)^b^	0.006^c^ (0.0008)^b^	0.09 (0.001)	0.01 (0.006)
GCV_SDe_ ^d^		0.087 (0.004)^b^		

^a^The conventional analysis has no permanent sow effect, since there is only a single observation per sow

^b^Standard errors obtained based on calculations from Mulder et al. [82]

^c^Heritability estimated at the level of squared phenotype: *h*_v_^2^ = *σ*_av_^2^/(2*σ*_P_^4^ + 3(*σ*_av_^2^ + *σ*_pev_^2^)) [70]

^d^Genetic coefficient of variation at residual standard deviation level, i.e. the genetic standard deviation in residual standard deviation divided by the mean residual standard deviation of the trait: ${\mathrm{GCV}}_{\mathrm{SDe}}=\frac{\sigma_{\mathrm{av}}\left({\sigma}_{\mathrm{e}}\right)}{\overline{\sigma_{\mathrm{e}}}}\approx {\scriptscriptstyle \frac{1}{2}}{\sigma}_{\mathrm{av}}$ [1]

**Table 3:** Correlation estimates (with SE) between the random effects on the level and the variance of litter size, estimated in Large White sows using a Double Hierarchical GLM (DHGLM) or conventional bivariate analysis

Effect	DHGLM	Conventional
Additive genetic	0.49 (0.04)	0.68 (0.04)
Permanent sow effect/Residual^a^	−0.83 (0.02)	−0.12 (0.007)

^a^The conventional analysis has no permanent sow effect, since there is only a single observation per sow. The correlation was estimated between residuals in two parts of the model

To show the potential response to selection we propose to use the Genetic Coefficient of Variation on the standard deviation level (GCV_SDe_, i.e. the genetic standard deviation in residual standard deviation divided by the mean residual standard deviation of the trait (see *Methods* section for more details). The GCV_SDe_ for varTNB in this study is 0.09, which is slightly lower than in previous studies focusing on litter size variation in pigs (0.10–0.15; [34, 41]), as reviewed by Hill and Mulder [1]. Nonetheless, the GCV_SDe_ reported here indicates sufficient potential for selection to reduce variation in TNB. By assuming that in an efficient breeding program a response of ~1 genetic standard deviation per generation can be achieved, the GCV_SDe_ of 0.09 indicates that the genetic standard deviation (SD) of TNB can be reduced by 9 % in one generation.

Table 3 shows the genetic correlations between random effects in the level and variance part of the model. The additive genetic correlation between TNB and varTNB is positive and moderate (0.49). This correlation is unfavorable, and indicates that sows with genetically large litters tend to have more variation in litter size. The correlation between the permanent sow effects on TNB and varTNB has the opposite sign: −0.83. This indicates that non-genetic (environmental) disturbances decrease the mean of TNB, with simultaneous increase in the variance of this trait.

To investigate further the large difference between the permanent and genetic correlations obtained with the DHGLM, we also performed a conventional bivariate analysis. To compare methods, models need to be on the same scale. A DHGLM takes a logarithm of residual variance in exponential model. Thus, in the conventional analysis we used mean TNB and the log-transformed variance of mean TNB (log(var(TNB))) per sow. The estimated additive genetic variances for mean TNB and for log(var(TNB)) were similar to values obtained from the DHGLM (Table 2). The conventional bivariate analysis yields also correlations between additive genetic effects and residuals of mean TNB and log(var(TNB)) (Table 3). (Note that the conventional analysis has no permanent sow effect, since there is only a single observation per sow.) The estimated additive genetic correlation was 0.68, whereas the residual correlation was–0.12. The genetic correlation has the same sign as the one from the DHGLM, but is slightly different in magnitude. The residual correlation in the conventional analysis has the same sign as the permanent environment correlation in the DHGLM, but is much closer to zero. When considering the covariances rather than the correlations, the residual covariance from the conventional analysis (−0.82) exceeds the permanent covariance from the DHGLM (−0.27). In the DHGLM, we assumed that the residuals are independent from each other. Hence, in the DHGLM, the permanent covariance has to account fully for non-genetic covariance between TNB and varTNB, which probably causes the extremely negative correlation between permanent effects.

Felleki et al. [35] reported an additive genetic correlation of–0.6 between TNB and varTNB, which has the opposite sign to the value reported here. The model used by Felleki et al. [35], however, did not include a covariance for permanent sow effect. When this covariance is not included, the model does not separate the effects properly. When the permanent covariance was omitted in our study, the additive genetic correlation had a negative value of–0.57. To account fully for all existing effects it is necessary to include the covariance structure between both permanent and additive genetic effects in the two parts of the model.

### Significant associations for TNB and varTNB

**Fig. 1:**
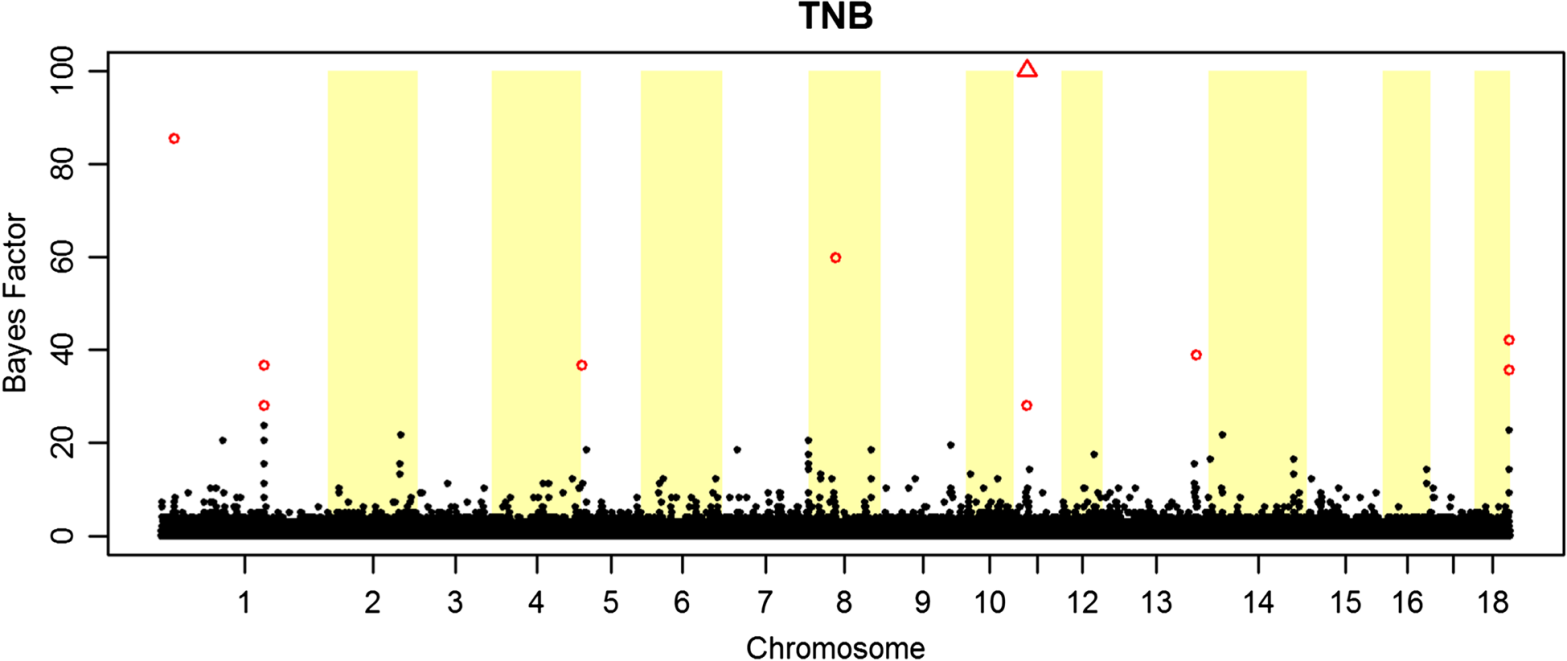
Genome-wide association for litter size (TNB) in 2,351 purebred boars and sows from a Large White pig population. Red circles indicate SNPs with BF ≥30, red triangles indicate SNP with BF ≥100 and black dots indicate SNPs with BF <30

**Fig. 2:**
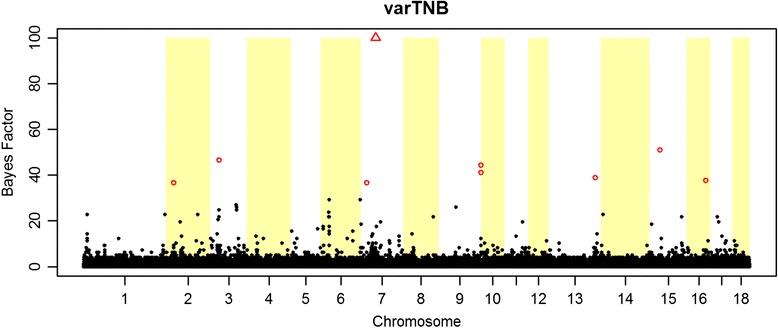
Genome-wide association for variation in litter size (varTNB) in 2,067 purebred boars and sows from a Large White pig population. Red circles indicate SNPs with BF ≥30, red triangles indicate SNP with BF ≥100 and black dots indicate SNPs with BF <30

In total, 10 significant SNPs were detected for TNB (Fig. 1) and 9 SNPs for varTNB (Fig. 2). Associations found for TNB where located mostly on the same *Sus scrofa* chromosomes (SSC) as reported in previous GWAS for this trait [25]. Since this is the first GWAS to report SNPs for variance of litter size in pigs, there are no studies available for comparison.

**Fig. 3:**
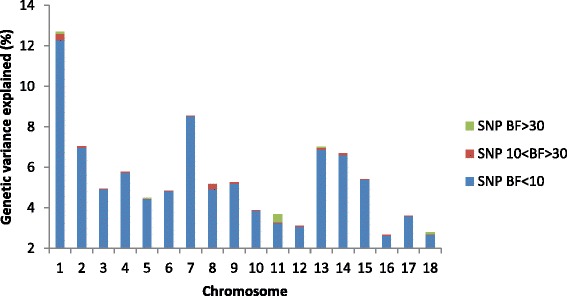
Percentage of genetic variance of litter size (TNB) explained per chromosome by significant SNPs with Bayes Factor (BF) above or equal to 30 (SNP BF ≥ 30), SNPs with BF equal or larger than 10 but lower than 30 (SNP 10 < BF > 30), and non-significant SNPs with BF below 10 (SNP BF < 10)

**Fig. 4:**
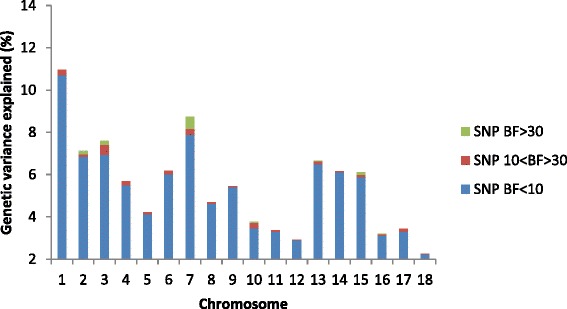
Percentage of genetic variance of litter size variability (varTNB) explained per chromosome by significant SNPs with Bayes Factor (BF) above or equal to 30 (SNP BF ≥ 30), SNPs with BF equal or larger than 10 but lower than 30 (SNP 10 < BF > 30), and non-significant SNPs with BF below 10 (SNP BF < 10)

Overall, the significant SNPs explained 0.83 % of the total genetic variance in TNB, and 1.44 % of the genetic variance in varTNB (Tables 4 and 5). The SNPs reported on SCC11 for TNB and all the SNPs for varTNB are the first SNPs reported for those traits in pigs. The chromosomes with the most variance explained were SSC11 for TNB and SSC7 for varTNB (Figs 3 and 4). On SSC11, ASGA0050328 associated with TNB explained 0.36 % of the total genetic variance. The estimated allele substitution effect at this locus was 0.105 piglets (Table 4). Previous studies that detected QTL for TNB, reported percentage of phenotypic variance explained by markers, rather than genetic variance, on the level between 0.3 % to 15 % [42–45], so higher than in this study. On SSC7, INRA0025193 explained 0.5 % of the genetic variance for varTNB. The allele substitution effect at this locus was 2.3 % of the mean value of varTNB (Table 5; note that values are given on log-variance scale). The small proportion of genetic variance explained by the significant associations indicates that both litter size and its variation are highly polygenic traits.

**Table 4:** Significant SNPs per *Sus scrofa* chromosome (SSC) associated with litter size and detected in boars and sows from a Large White pig population. Description of significant regions includes: minor allele frequency (MAF), allele substitution effect and the Bayes Factor (BF) as an indication for significance (only SNP with BF ≥ 30 are reported)

SSC	Significant SNP	Position (Mb)	MAF	Allele subs. effect^a^	BF	Gen. var. expl. by SNP (%)
1	ALGA0001244	17.28	0.30	0.052	85.4	0.10
1	ASGA0005117	182.38	0.48	0.039	36.6	0.07
1	ALGA0006771	182.42	0.48	0.034	30.1	0.05
5	ASGA0023713	1.38	0.35	0.034	36.6	0.04
8	ASGA0097249	42.52	0.42	0.021	59.9	0.02
11	ASGA0050328	23.81	0.26	0.105	295.5	0.36
11	MARC0020561	23.87	0.45	0.030	30.1	0.04
13	ASGA0059543	192.72	0.25	0.042	38.8	0.06
18	ALGA0098906	58.86	0.29	0.038	35.5	0.05
18	INRA0056201	58.88	0.29	0.036	42.1	0.04

^a^Allele substitution effects were estimated as $\alpha =\sqrt{\sigma_a^2{(2pq)}^{-1}}$ *,* where *σ*_*a*_^2^ is the genetic variance explained by the SNP, and p and q are the frequencies of the two alleles [83].

**Table 5:** Significant SNPs per *Sus scrofa* chromosome (SSC) associated with variation in litter size and detected in boars and sows from a Large White pig population. Description of significant regions includes: minor allele frequency (MAF), allele substitution effect, the Bayes Factor (BF) as an indication for significance (only SNP with BF ≤ 30 are reported) and overview of previously reported QTL for reproduction traits in pigs (based on PigQTLdb; February, 2015)

SSC	Most sign. SNP	Position (Mb)	MAF	Allele subs. effect^a^	BF	Gen. var. expl. by SNP (%)	Overview of QTL reported within region
2	ALGA0106652	27.17	0.44	0.011	36.6	0.11	
3	MARC0056802	28.40	0.27	0.016	46.4	0.20	corpus luteum number [44] plasma concentration of FSH [44]
7	INRA0025193	43.76	0.48	0.023	167.2	0.50	corpus luteum number [84] birth weight [46, 85–87]
7	ASGA0031511	17.47	0.20	0.010	36.6	0.06	corpus luteum number [84] female age at puberty [44]
10	H3GA0055101	0.05	0.47	0.011	44.2	0.12	number of stillborn [43]
10	MARC0015344	0.06	0.47	0.012	41.0	0.15	
13	DRGA0013310	194.39	0.32	0.011	38.8	0.10	corpus luteum number [84]
15	MARC0077161	35.59	0.39	0.010	50.9	0.10	corpus luteum number [84]
16	DRGA0016314	73.39	0.31	0.009	37.7	0.07	birth weight [46]

^a^Allele substitution effects were estimated as $\alpha =\sqrt{\sigma_a^2{(2pq)}^{-1}}$ *,* where *σ*_*a*_^2^ is the genetic variance explained by the SNP, and p and q are the frequencies of the two alleles [83]. The estimated allele substitution effects refer to the log-variance

The estimated genetic correlation between TNB and its variation (0.49) could suggest presence of pleiotropic effects or SNPs in linkage disequilibrium (LD). However, we did not identify overlap in regions significant for TNB and varTNB (Figs 1 and 2). Only on SSC13, SNPs for both traits are located close to each other (Tables 4 and 5), but they are not in LD.

### Candidate genes and QTL associated with TNB

The two regions detected on SSC11 are the first SNPs associated with TNB on this chromosome. Thus far, no other study available in PigQTLdb (based on February 2015 search) reported significant associations for TNB on SSC11. Only one study reported QTL for a reproduction trait within the region of the most significant SNP (ASGA0050328) for TNB, which was a QTL for number of teats [46].

No candidate genes could be identified within the region of ±50kbp around ASGA0050328 (Ensembl Sscrofa 10.2; February 2015). The nearest candidate gene named *ENOX1* was found at 24.16-24.48 Mb. One of the SNPs associated with TNB (with BF = 10.2) was located in this region. The *ENOX1* is a protein coding gene from the ecto-CNOX family being part of electron transport pathways associated with mitochondrial membranes [47]. Functions of *ENOX1* are cellular defense and growth as well as cell survival [47]. The functions of *ENOX1* indicate that this gene might be a new region relevant for TNB in pigs.

In addition, the region detected on SSC18 (58.86-58.88 Mb) shows relevance for TNB in pigs. Three QTL related to reproduction traits were previously described within this region ([25]; February 2015). Those QTL were for: TNB [43], corpus luteum number [48], and gestation length [43]. Moreover, we have identified a possible candidate gene from the galectin family named *LGALS8* within the detected region. The *LGALS8* is widely expressed in tumoral tissues and seems to be involved in integrin-like cell interactions, cell-cell adhesion, cell-matrix interaction and growth regulation [49].

### Candidate genes and QTL for variability of TNB

Quantitative trait loci associated with phenotypic variability are defined in the literature as vQTL [50]. In this study, the SNPs associated with varTNB are the first vQTL reported in pigs. Detected SNPs for varTNB were located at the positions of several known QTL related to reproduction traits in pigs. Those QTL are summarized in Table 5.

Within the region of the most significant SNP (INRA0025193) for varTNB at 43.76 Mb on SSC7, one candidate gene was located named *CUL9* (SSC7:43,72-43,76 Mb) *CUL9* is a cytoplasmic anchor protein in complex associated with p53 [51, 52]. The p53 is a protein, which regulates the cycle of the cell and acts as a tumor suppressor [53]. The *CUL9* is controlling the localization and the function of p53 in the cell [51, 52]. Even though *CUL9* was not yet described in swine, its functions can be important in affecting litter size variability in pigs, especially since *CUL9* is expressed in embryonic, placental, and uterus tissues in the human [54].

Two more SNPs on SSC7 associated with varTNB (with BF 10.2 and 17.5) were located within the regions of two other possible candidate genes already described in swine: *HSPCB* (SSC7: 45.11-45.12 Mb) and *VEGFA* (SSC7: 44.46-44.47 Mb). The first gene belongs to the *Sus scrofa* heat shock protein family. This protein family is referred to as molecular chaperones since they are activated under various stress condition, such as heat [55], hyperthermia [56], and inflammation [57]. Their function is to maintain proper folding of the proteins within a cell as well as re-folding denatured proteins post-stress [58, 59]. Known in *Drosophila* and *Arabidopsis* as *Hsp90*, it is well described as a gene stabilizing developmental and morphological traits. The *Hsp90* was describe to buffer environmental (e.g. heat shock, inflammation) and genetic (e.g. unfavorable mutations) factors, resulting in low variation of developmental and morphological traits [22–24]. Even though *Hsp90* was shown to be one of many genes with buffering effects [60, 61], it is a very promising candidate gene detected for varTNB in this study. The second gene, named *VEGFA*, is a vascular endothelial growth factor. A *VEGFA* is a protein mediator growth factor activated in angiogenesis and vasculogenesis in the fetus (and adult) [62] as well as in endothelial cell growth [63]. These two candidate genes detected on SSC7 are highly relevant, since those genes affect the response of the pig to environmental and stress factors (*HSPCB*) and provide the vascular network to the placenta (*VEGFA*).

### Implications for pig breeding

An important aim in pig production is to obtain a high number of slaughter pigs per sow per year [26, 64, 65]. Therefore, in pig breeding, genetic selection continues to increase litter size. The annual genetic trend for litter size in different pig breeding programs was shown to be +0.16 [29, 66], +0.25 [67], and even up to +0.5 [68] piglets per year on average. Next to genetic variation in litter size, there is considerable residual variation in this trait, both between sows and between parities within a sow.

We showed that residual variance in litter size has a genetic component, and can thus be changed by selection. The results of our study also show presence of an unfavorable positive genetic correlation between litter size and its variation. So far, main inefficiency in fecundity was the presence of too small litters. More and more, however, too large litters are becoming the prevailing problem. Therefore, simultaneous selection of litter size and it variation is necessary to achieve a higher mean litter size and at the same time a lower variance in litter size. This is important for pig production, since both small and oversized litters can be detrimental for farm economy. Although the genetic correlation was 0.49, we did not identify overlap in regions significant for TNB and varTNB (Tables 4 and 5).

Current breeding programs can use the knowledge of this study in the genomic evaluation of selection candidates. Genomic selection can greatly increase accuracy of selection also for non-phenotyped individuals. This is beneficial for traits such as litter size variability, which has low heritability and is recorded only long after the moment of selection of the candidate.

## Conclusions

To our knowledge, this is the first study reporting SNPs for TNB on SSC11 and first SNPs associated with varTNB in pigs. In total, 10 SNPs were detected for TNB and 9 SNPs for varTNB. The most significant SNP for TNB was detected on SSC11. A possible candidate gene for TNB on SSC11, named *ENOX1*, is involved in cell growth and survival. Also on SSC18, another possible candidate gene for TNB is located, named *LGALS8.* Two genes located on SSC7 (*HSPCB* and *VEGFA*) are the most promising candidate genes identified for varTNB. The *HSPCB* is coding the heat-shock protein involved in buffering environmental and genetic factors, whereas *VEGFA* is activated in angiogenesis and vasculogenesis in the fetus. We also found a positive genetic correlation between TNB and its variation. This indicates that in breeding practice simultaneous selection of those traits is necessary to achieve a higher mean litter size and lower variance of this trait. The SNPs detected in this study can be used as an indication for regions in the *Sus scrofa* genome involved in maintaining low variability of litter size, but further studies are needed to confirm causative mutations.

## Methods

### Phenotypes

Data for this study were collected between February 1998 and July 2014. In total 264,419 litter size (total number born, TNB) observations were available from 69,549 Large White sows. Litters were kept in the data if they contained at least 4 piglets in TNB (1,331 litters removed), whereas litters of 27 piglets or larger were all considered “27” (43 litters). Most sows had repeated observations; only 15,379 sows had a single observation. Number of parities recorded per sow varied between 1 and 16, with an average of 3.8 per sow, and with 974 sows with 10 or more parities recorded. The parities 10 and higher were put on the same level of explanatory variable in the model (2,512 litters). After data editing 263,088 litters from 69,238 Large White sows remained for the analysis. The average TNB in edited data was 13.5 (±3.5). The pedigree was traced back 5 generations if available and consisted of 83,571 animals.

### Conventional univariate and bivariate analysis of litter size and its variance

The conventional univariate analysis of TNB was performed in ASReml 2.0 [69] using the following model:

$$ \mathbf{y}=\mathbf{X}\mathbf{b}+\mathbf{Z}\mathbf{a}+\mathbf{U}\mathbf{p}\mathbf{e}+\mathbf{e}, $$

where **y** is a vector of observation on TNB in the litter; **b** is a vector of fixed effects (parity of the sow and farm_year_season of the farrowing) on **y**; **a** is a vector of random additive genetic effects on **y**, with **a** ~ *N*(**0**, **A***σ*_a_^2^); **pe** is a vector of random non-genetic permanent sow effects on **y**, with **pe** ~ *N*(**0**, **A***σ*_pe_^2^); and **e** is a vector of residual, with **e** ~ *N*(**0**, **I**_e_*σ*_e_^2^).

The variance component estimates obtained with the conventional univariate analysis were used as starting values in the Double Hierarchical Generalized Linear model (DHGLM).

The conventional bivariate analysis of litter size and its variation was performed on average TNB per sow (meanTNB) and log-transformed variance of TNB per sow (log(var(TNB))). The log-transformation was necessary to obtain results on the same scale in the conventional analysis and in the DHGLM. In the conventional bivariate analysis, only sows with 3 or more parities were used to allow proper estimation of the variance. In total, observations on 43,490 sows were used. The bivariate analysis was performed in ASReml 2.0 [69] with model:

$$ \left[\begin{array}{c}\hfill {\mathbf{y}}_{\mathrm{mean}}\hfill \\ {}\hfill {\mathbf{y}}_{\mathrm{v}}\hfill \end{array}\right]=\left[\begin{array}{cc}\hfill \mathbf{X}\hfill & \hfill \mathbf{0}\hfill \\ {}\hfill \mathbf{0}\hfill & \hfill {\mathbf{X}}_{\mathrm{v}}\hfill \end{array}\right]\left[\begin{array}{c}\hfill {\mathbf{b}}_{\mathrm{mean}}\hfill \\ {}\hfill {\mathbf{b}}_{\mathrm{v}}\hfill \end{array}\right]+\left[\begin{array}{cc}\hfill {\mathbf{Z}}_{\mathrm{mean}}\hfill & \hfill \mathbf{0}\hfill \\ {}\hfill \mathbf{0}\hfill & \hfill {\mathbf{Z}}_{\mathrm{v}}\hfill \end{array}\right]\left[\begin{array}{c}\hfill {\mathbf{a}}_{\mathrm{mean}}\hfill \\ {}\hfill {\mathbf{a}}_{\mathrm{v}}\hfill \end{array}\right]+\left[\begin{array}{c}\hfill {\mathbf{e}}_{\mathrm{mean}}\hfill \\ {}\hfill {\mathbf{e}}_{\mathrm{v}}\hfill \end{array}\right], $$

where **y**_mean_ is a vector of observations on average TNB per sow and **y**_v_ is a vector of the log-transformed variance of meanTNB; **b**_mean_ and **b**_v_ are vectors of fixed effects of farm_year_season of the farrowing on **y**_mean_ and **y**_v_; **a**_mean_ and **a**_v_ are vectors of random additive genetic effects on **y**_mean_ and **y**_v_, with $$ \left[\begin{array}{c}\hfill {\mathbf{a}}_{\mathrm{mean}}\hfill \\ {}\hfill {\mathbf{a}}_{\mathrm{v}}\hfill \end{array}\right]\sim \mathrm{N}\left(\mathbf{0},\left[\begin{array}{cc}\hfill {\sigma}_{{\mathrm{a}}_{\mathrm{mean}}}^2\hfill & \hfill {\sigma}_{{\mathrm{a}}_{\mathrm{mean}},\mathrm{a}\mathrm{v}}\hfill \\ {}\hfill {\sigma}_{{\mathrm{a}}_{\mathrm{mean}},\mathrm{a}\mathrm{v}}\hfill & \hfill {\sigma}_{\mathrm{a}\mathrm{v}}^2\hfill \end{array}\right]\otimes \mathbf{A}\right) $$; and **e** and **e**_v_ vectors of residuals, with $$ \left[\begin{array}{c}\hfill {\mathbf{e}}_{\mathrm{mean}}\hfill \\ {}\hfill {\mathbf{e}}_{\mathrm{v}}\hfill \end{array}\right]\sim \mathrm{N}\left(\begin{array}{c}\hfill \mathbf{0}\hfill \\ {}\hfill \mathbf{0}\hfill \end{array},\left[\begin{array}{cc}\hfill {\mathbf{W}}^{*}{\upsigma}_{{\mathrm{e}}_{\mathrm{mean}}}^2\hfill & \hfill 0\hfill \\ {}\hfill 0\hfill & \hfill {\mathbf{W}}^{*}{\upsigma}_{\mathrm{e}\mathrm{v}}^2\hfill \end{array}\right]\right) $$, where W^*^ is the weighting factor based on the number of litters the sow had. The conventional bivariate analysis has no permanent sow effect, since there is only a single observation per sow.

### Estimation of residual variance of litter size

Estimation of residual variance was performed on the full data set. A Double Hierarchical Generalized Linear model (DHGLM) as presented by Rönnegård et al. [2] allows estimation of variance components of residual variance in ASReml 2.0 [69]. Felleki et al. [35] extended the model so that a bivariate linear mixed model can be used for the level (TNB) and the variance (TNB variation, varTNB) component of the model. In Rönnegård et al. [2], the response variable in the variance model was calculated as $\log \left({\varphi}_i\right)= \log \left(\frac{e_i^2}{1-{h}_i}\right)$, where *e*_*i*_^2^ is the squared residual from the level part of the model for observation *i,* and *h*_*i*_ is the leverage, being the diagonal element of the hat matrix of **y** corresponding to observation *i*. A log link function was used, because $\frac{e_i^2}{1-{h}_i}$ is *χ*^2^-distributed with one degree of freedom. Felleki et al. [35] showed that instead of using a log link function, $\log \left(\frac{e_i^2}{1-{h}_i}\right)$ can be linearized using the Taylor expansion of the first order by calculating the response variable ${\psi}_i= \log \left({\widehat{\sigma}}_{e_i}^2\right)+\frac{\frac{e_i^2}{1-{h}_i}-{\widehat{\sigma}}_{e_i}^2}{{\widehat{\sigma}}_{e_i}^2}$. This enables using a bivariate linear mixed model, where ${\widehat{\sigma}}_{e_i}^2$ is the predicted residual variance for observation *i* and *ψ* is a vector with the response variable in the variance part of the model. Note that *ψ*_*i*_ is a linearized working variable for log(*φ*_*i*_). The DHGLM is then as follows:

$$ \left[\begin{array}{c}\hfill \mathbf{y}\hfill \\ {}\hfill \boldsymbol{\uppsi} \hfill \end{array}\right]=\left[\begin{array}{cc}\hfill \mathbf{X}\hfill & \hfill \mathbf{0}\hfill \\ {}\hfill \mathbf{0}\hfill & \hfill {\mathbf{X}}_{\mathrm{v}}\hfill \end{array}\right]\left[\begin{array}{c}\hfill \mathbf{b}\hfill \\ {}\hfill {\mathbf{b}}_{\mathrm{v}}\hfill \end{array}\right]+\left[\begin{array}{cc}\hfill \mathbf{Z}\hfill & \hfill \mathbf{0}\hfill \\ {}\hfill \mathbf{0}\hfill & \hfill {\mathbf{Z}}_{\mathrm{v}}\hfill \end{array}\right]\left[\begin{array}{c}\hfill \mathbf{a}\hfill \\ {}\hfill {\mathbf{a}}_{\mathrm{v}}\hfill \end{array}\right]+\left[\begin{array}{cc}\hfill \mathbf{U}\hfill & \hfill \mathbf{0}\hfill \\ {}\hfill \mathbf{0}\hfill & \hfill {\mathbf{U}}_{\mathrm{v}}\hfill \end{array}\right]\left[\begin{array}{c}\hfill \mathbf{p}\mathbf{e}\hfill \\ {}\hfill \mathbf{p}{\mathbf{e}}_{\mathrm{v}}\hfill \end{array}\right]+\left[\begin{array}{c}\hfill \mathbf{e}\hfill \\ {}\hfill {\mathbf{e}}_{\mathrm{v}}\hfill \end{array}\right], $$

where **y** is a vector of observations on TNB in the litter and *ψ* is a vector of response variables for the variance part of DHGLM; the residuals **e** and **e**_v_ are assumed to be independent and normally distributed, but with heterogeneous variances across the observations; **b** and **b**_v_ are vectors of fixed effects (parity of the sow and farm_year_season of the farrowing) on **y** and **ψ**; **a** and **a**_v_ are vectors of random additive genetic effects on **y** and **ψ**, with $$ \left[\begin{array}{c}\hfill \mathbf{a}\hfill \\ {}\hfill {\mathbf{a}}_{\mathrm{v}}\hfill \end{array}\right]\sim \mathrm{N}\left(\mathbf{0},\left[\begin{array}{cc}\hfill {\sigma}_{\mathrm{a}}^2\hfill & \hfill {\sigma}_{\mathrm{a},\mathrm{a}\mathrm{v}}\hfill \\ {}\hfill {\sigma}_{\mathrm{a},\mathrm{a}\mathrm{v}}\hfill & \hfill {\sigma}_{\mathrm{a}\mathrm{v}}^2\hfill \end{array}\right]\otimes \mathbf{A}\right) $$; **pe** and **pe**_v_ are vectors of random non-genetic permanent sow effects on **y** and **ψ**, with $$ \left[\begin{array}{c}\hfill \mathbf{p}\mathbf{e}\hfill \\ {}\hfill \mathbf{p}{\mathbf{e}}_{\mathbf{v}}\hfill \end{array}\right]\sim \mathrm{N}\left(\mathbf{0},\left[\begin{array}{cc}\hfill {\upsigma}_{\mathrm{pe}}^2\hfill & \hfill {\upsigma}_{\mathrm{pe},\mathrm{p}\mathrm{e}\mathrm{v}}\hfill \\ {}\hfill {\upsigma}_{{\mathrm{pe},\mathrm{p}\mathrm{e}}_{\mathrm{v}}}\hfill & \hfill {\upsigma}_{\mathrm{pe}\mathrm{v}}^2\hfill \end{array}\right]\otimes \mathbf{I}\right) $$; and **e** and **e**_v_ vectors of residuals, with $$ \left[\begin{array}{c}\hfill \mathbf{e}\hfill \\ {}\hfill {\mathbf{e}}_{\mathrm{v}}\hfill \end{array}\right]\sim \mathrm{N}\left(\begin{array}{c}\hfill \mathbf{0}\hfill \\ {}\hfill \mathbf{0}\hfill \end{array},\left[\begin{array}{cc}\hfill {\mathbf{W}}^{\hbox{-} 1}{\upsigma}_{\mathrm{e}}^2\hfill & \hfill 0\hfill \\ {}\hfill 0\hfill & \hfill {\mathbf{W}}_{\mathrm{v}}^{\hbox{-} 1}{\upsigma}_{\mathrm{e}\mathrm{v}}^2\hfill \end{array}\right]\right) $$, where $\mathbf{W}=\mathrm{diag}\left( \exp {\left(\widehat{\boldsymbol{\uppsi}}\right)}^{-1}\right)$ and ${\mathbf{W}}_{\mathrm{v}}=\mathrm{diag}\left(\frac{1-h}{2}\right)$ are expected reciprocals of the residual variance from the previous iteration, and σ_e_^2^ and σ_ev_^2^ are scaling variances, which are expected to be equal to 1 [12]. The predicted residual variances per observation $\exp \left(\widehat{\boldsymbol{\uppsi}}\right)$ are based on the estimated fixed and random effects for **ψ** in the previous iteration of the algorithm. The method iterates the bivariate model a number of times until convergence, since the residual variance part (varTNB) depends on the level part (TNB) of the model and vice versa. In this study, 22 iterations were needed.

A DHGLM uses the log-transformed variance of the trait. To help with practical interpretation of the results, two measures will be used: Genetic Coefficient of Variation at standard deviation level and the heritability for residual variance. The Genetic Coefficient of Variation on standard deviation level (GCV_SDe_, a measure of ability to respond to selection) was applied to transform the estimates from the variance to the SD level. The GCV_SDe_ is calculated as the genetic standard deviation in residual standard deviation divided by the mean residual standard deviation of the trait: ${\mathrm{GCV}}_{\mathrm{SDe}}=\frac{\sigma_{\mathrm{av}}\left({\sigma}_{\mathrm{e}}\right)}{\overline{\sigma_{\mathrm{e}}}}\approx {\scriptscriptstyle \frac{1}{2}}{\sigma}_{\mathrm{av}}$, where σ_av_ is the genetic SD in residual variance. The GCV_SDe_ shows the proportional change in residual SD, when the residual variance would be changed by one unit σ_av_. This allows seeing the magnitude of potential response to selection in the SD of TNB. Note that in Hill and Mulder [1] the GCV was expressed at the level of the residual variance; the GCV at the level of residual variance is twice the GCV_SDe._ The literature on genetic analyses of residual variance defines also the heritability for residual variance at the level of the squared phenotype (*h*_v_^2^), which is equal to the genetic variance in residual variance as a proportion of the phenotypic variance of P^2^ and equals: *h*_v_^2^ = *σ*_av_^2^/(2*σ*_P_^4^ + 3(*σ*_av_^2^ + *σ*_pev_^2^)) [70]. The *h*_v_^2^ is a measure of reliability of EBV obtained on a single observation per animal; in contrast to classical heritability, it does not reflect the potential of the trait to respond to selection.

### Using deregressed EBV for litter size and litter size variability

In this study, a large number of phenotypic observations for TNB were available, but much lower number of sows and boars was genotyped (see *Genotypes* below). In addition, the boars had only observations through their daughters’, sisters’ and mothers’ performance. The use of deregressed EBV of animals instead of their phenotype is expected to give more reliable results in genome-wide association study; since accounting for offspring and parents information increases the power of the GWAS [71]. Therefore, for the optimal use of the entire data in the GWAS, the estimated breeding values (EBV) obtained with DHGLM were deregressed. The deregressed EBV were used as y-variables in the GWAS (see below).

The EBV were deregressed following the methodology of Garrick et al. [72]. First, the parent average was subtracted from an individual’s EBV to avoid double counting because of various information sources and complex family structure. Thus, the sow’s (or boar’s) deregressed EBV contained only the information on own and progeny performance. Second, for calculation of deregressed EBV the reliability of EBV was required. Reliability was calculated following the equation [69]:

$$ {r}^2=1-\frac{s_i^2}{\left(1+{f}_i\right){\sigma}_a^2}, $$

where s_i_ is the standard error reported for the EBV of the i^th^ individual; f_i_ is the inbreeding coefficient of the i^th^ individual; 1 + f_1_ is the diagonal element of the additive genetic relationship matrix and *σ*_*a*_^2^ is the additive genetic variance. Finally, Garrick et al. [72] showed that deregressed EBV have heterogeneous variances, which can be accounted for using weights (w). The weight was estimated based on the reliability of calculated deregressed EBV.

Deregressed EBV were obtained based on EBV from two univariate analyses of the traits TNB and varTNB using the results from the final iteration of the DHGLM (see *Estimation of residual variance*). The EBV from univariate analysis were used to avoid EBV for one trait to be affected by the other trait in the bivariate analysis. The deregressed EBV obtained with the DHGLM for TNB, were also compared with those from the conventional bivariate analysis. The correlation between deregressed EBV for TNB from both methods was 0.988.

### Genotypes

Genotypes were available for 2,679 Large White sows and 426 boars. All animals were genotyped with the Illumina PorcineSNP60 Beadchip. Samples of blood, hair and ear punches used to extract DNA were collected in the process of routine procedure within the breeding program and as such did not require an approval from Animal Care and Use Committee. Quality control removed SNPs with GenCall score <0.15, minor allele frequency <0.01, as well as SNPs from the sex chromosome (low number of animals had sex chromosome genotyped) or with unknown position on build 10.2. After quality control, 40,969 out of 64,232 genotyped SNPs remained in the data set. In addition, animals were removed from the data set if their call rate was <95 % and if pedigree or genotype led to many Mendelian inconsistencies [73].

The deregressed EBV for litter size variability have overall low reliabilities, due to the low heritability of that trait. To maintain a sufficient number of genotyped animals for the genome-wide association study (GWAS), a threshold of 0.05 was used as an acceptable reliability of the deregressed EBV of the animal. Nonetheless, this caused a difference in number of animals used in GWAS for TNB and varTNB. Subsequently, 2,351 animals remained in the set with litter size observations and 2,067 in the set for residual variance of litter size.

### Statistical analyses used for GWAS–Bayesian Variable Selection method

Multi-SNP genome-wide association was performed using a Bayesian Variable Selection method [74], which estimates the effect of all markers simultaneously. The analysis was performed in Bayz [75]. The fitted model was:

$$ \mathrm{y} = \mu + {\mathrm{X}}_{\mathrm{b}}\upbeta + \mathrm{e}, $$

where **y** is an n-vector of deregressed EBV for the litter size or its variation on n animals; **μ** is an n-vector equal to the mean; **X**_**b**_ is a matrix with dimensions n by p, where p SNPs are coded as 0, 1, 2 copies of specific allele vector; and β is a p-vector with the markers effects fitted as random effects; *e* is an n-vector of weighted random residual effects assumed to be normally distributed N(0, *σ*_e_^2^**W**_**t**_), where **W**_**t**_ is a diagonal matrix with w_t1_,…,w_tn_ elements. On the marker effect, the Bernoulli distribution was applied:

$$ \beta \sim \left\{\begin{array}{c}\hfill N\left(0,{\sigma}_{g_0}^2\right)\mathrm{with}\ \mathrm{probability}:\ {\pi}_0\hfill \\ {}\hfill N\left(0,{\sigma}_{g_1}^2\right)\mathrm{with}\ \mathrm{probability}:\ {\pi}_1\hfill \end{array}\right. $$

where the first distribution refers to the null distribution and it is assumed that the SNPs have small effect (${\sigma}_{g_0}^2$); the second distribution refers to the SNPs that are assumed to have a large effect, which explain a large part of variance (${\sigma}_{g_1}^2$) of the analyzed traits. In this study a relatively strict prior was selected of π_1_ = 0.001, meaning that on average only 1 in 1,000 SNPs will be in the second distribution in each cycle. This allowed only ~41 SNPs per cycle to have a large effect on the traits. To secure that all the SNPs were used, 500,000 MCMC cycles were performed. Selecting a stringent prior provides a more precise distinction between SNPs with large and small effects on the trait [76, 77].

A Metropolis-Hastings sampler was applied to get good convergence which was assessed by visual inspection of the trace and with Gelman and Rubin’s convergence diagnostic based on deviance [78] using the R package CODA [79].

The GWAS was also repeated with a less strict prior of 0.005 and a larger step in the Metropolis-Hastings sampler (0.004 instead of 0.003), which yielded the same results as the analysis with prior of 0.001.

### Identification of significant SNPs

The Bayes Factor (BF) was calculated for each SNP to determine the significant associations:

$$ \mathrm{B}\mathrm{F}=\frac{{\widehat{p}}_i/\left(1-{\widehat{p}}_i\right)}{\pi_1/{\pi}_0}, $$

where π_1_ and π_0_ are the prior probabilities and ${\widehat{p}}_i$ is the posterior probability of the fraction of times the SNP was in the distribution with large effect. Following the definitions of Kass and Raftery [80], the SNPs with BF > 30 are described as “very strong” association and with BF > 150 as “decisive”. The variance explained by significant SNP was estimated as a fraction of the total genetic variance explained by all SNPs. The candidate gene search was performed with software BIOMART available in Ensembl Sscrofa 10.2 [81] by entering position of a SNP.

### Availability of supporting data

The dataset used in this study is available upon request. Contact Egbert Knol by e-mail: Egbert.Knol@topigsnorsvin.com. Furthermore, the SNPs detected within this study are submitted to an open access animal QTL database – Pig QTL database (http://www.animalgenome.org/QTLdb/pig.html).
